# Lower-Limb Motor Imagery Recognition Prototype Based on EEG Acquisition, Filtering, and Machine Learning-Based Pattern Detection

**DOI:** 10.3390/s25206387

**Published:** 2025-10-16

**Authors:** Sonia Rocío Moreno-Castelblanco, Manuel Andrés Vélez-Guerrero, Mauro Callejas-Cuervo

**Affiliations:** Software Research Group, Universidad Pedagógica y Tecnológica de Colombia, Tunja 150002, Colombia; sonia.moreno01@uptc.edu.co (S.R.M.-C.); manuel.velez@uptc.edu.co (M.A.V.-G.)

**Keywords:** Brain–Computer Interface (BCI), electroencephalography (EEG), motor imagery (MI), lower-limb, signal processing, filtering, Butterworth, Savitzky–Golay, artificial intelligence, machine learning, Random Forest, pattern detection

## Abstract

Advances in brain–computer interface (BCI) research have explored various strategies for acquiring and processing electroencephalographic (EEG) signals to detect motor imagery (MI) activities. However, the complexity of multichannel clinical systems and processing techniques can limit their accessibility outside specialized centers, where complex setups are not feasible. This paper presents a proof-of-concept prototype of a single-channel EEG acquisition and processing system designed to identify lower-limb motor imagery. The proposed proof-of-concept prototype enables the wireless acquisition of raw EEG values, signal processing using digital filters, and the detection of MI patterns using machine learning algorithms. Experimental validation in a controlled laboratory with participants performing resting, MI, and movement tasks showed that the best performance was obtained by combining Savitzky–Golay filtering with a Random Forest classifier, reaching 87.36% ± 4% accuracy and an F1-score of 87.18% ± 3.8% under five-fold cross-validation. These findings confirm that, despite limited spatial resolution, MI patterns can be detected using appropriate AI-based filtering and classification. The novelty of this work lies in demonstrating that a single-channel, portable EEG prototype can be effectively used for lower-limb MI recognition. The portability and noise resilience achieved with the prototype highlight its potential for research, clinical rehabilitation, and assistive device control in non-specialized environments.

## 1. Introduction

In recent decades, the study of brain activity associated with motor control has undergone a significant transformation, driven by advances in neuroscience, biomedical engineering, and signal processing [1]. These developments have enabled applications in neuromotor rehabilitation, prostheses and exoskeleton control, as well as in interactive and training environments [1,2,3].

Key factors behind this transformation include advances in noninvasive signal acquisition and processing techniques, such as electroencephalography (EEG), along with the refinement of artificial intelligence (AI)-based algorithms capable of extracting and classifying neural patterns with increasing precision [4,5,6,7].

Traditionally, EEG signal processing in Brain–Computer Interface (BCI) applications has relied on general-purpose hardware such as laptops or desktop computers, which provide sufficient computational power for filtering and classification algorithms [8,9]. However, dependence on this equipment restricts portability and integration, underscoring the need for lighter solutions that balance efficiency with ease of deployment [10]. In response, portable hardware combined with simpler yet effective preprocessing algorithms has expanded BCI accessibility beyond specialized environments [2,11,12]. BCIs enable direct communication between brain activity and external systems [13,14], with major applications in rehabilitation and assistive device control through motor intention detection [1]. While clinical multichannel systems offer high spatial resolution, portable systems prioritize accessibility and usability [7]. Nevertheless, many remain constrained by high cost, installation complexity, or reduced signal accuracy and robustness [15].

A gap persists in developing BCIs that combine portability and ease of use with sufficient accuracy for neuromotor rehabilitation and clinical research [16]. Current approaches often emphasize either spatial resolution with high-cost, multichannel systems [17] or accessibility with simplified setups, at the expense of performance [18,19]. Moreover, most studies have focused on upper-limb motor imagery, while lower-limb paradigms remain comparatively underexplored despite their importance for functional recovery and gait rehabilitation [20]. In addition, few works have systematically analyzed how hardware simplification impacts signal quality, classification accuracy, and robustness under realistic experimental conditions, which limits the translation of these systems to daily or clinical use.

Evidence regarding the reliability of minimal EEG configurations, such as single-channel portable devices for recognizing lower-limb motor imagery, remains limited and not yet well established in the literature. This gap also extends to the integration of lightweight preprocessing and machine learning algorithms optimized for embedded platforms, which are essential to achieve real-time, autonomous operation without reliance on external computers. This study, therefore, aims to contribute to addressing this gap by evaluating the feasibility of a low-cost, single-channel system as a preliminary step toward more accessible and portable BCI solutions [21].

A deeper understanding of EEG dynamics is essential to guide the design of portable BCIs, since the signal’s temporal characteristics and susceptibility to noise directly influence the performance of feature extraction and classification algorithms. EEG remains essential for motor imagery due to its temporal resolution and noninvasive nature [22]. It captures frequency variations linked to cognitive and motor states, mainly in the α, β, and γ bands, which are critical for detecting motor intention [23,24]. Standard processing involves filtering, normalization, and feature extraction to reduce noise and generate discriminative features for classification [25,26]. However, EEG is affected by noise, low signal-to-noise ratio, and inter-subject variability, which hinder recognition in simplified systems [27,28]. These challenges are greater in lower-limb studies, where signals show weaker amplitude and discriminability than upper-limb activity [29]. Robust filtering and advanced classification are therefore needed to improve reliability in portable, low-cost single-channel systems.

Building upon these considerations, this work presents the design, development, and preliminary validation of a proof-of-concept prototype for recognizing lower-limb motor imagery. The prototype acquires EEG signals, applies filtering, and detects patterns with machine learning to improve segmentation accuracy while reducing processing time.

A low-cost, portable implementation combines a single-channel EEG device with an embedded unit, enabling evaluation of simple acquisition with advanced processing to strengthen brain-state classification. This approach contributes to accessible and reliable solutions with applications in multiple domains, while also setting the ground for highlighting its innovative value within the biomedical sensors and neural engineering fields [30,31,32].

In this context, the present study highlights the originality of adopting a simplified single-channel EEG configuration to explore lower-limb motor imagery recognition. While multichannel systems remain the standard for achieving higher accuracy and richer data, our approach demonstrates that acceptable performance can also be obtained with reduced hardware complexity. By prioritizing portability and simplicity, the system aligns with ongoing efforts to design accessible BCI technologies and extends their applicability to resource-limited environments, rehabilitation contexts, and other real-world domains.

This work therefore contributes to bridging the gap between accessibility and accuracy by providing experimental evidence that even minimal EEG configurations, when properly processed, can yield viable recognition of lower-limb motor imagery. The remainder of the paper is organized as follows. Section 2 provides background. Section 3 details materials and methods, including prototype design and the experimental protocol. Section 4 reports the results. Section 5 discusses the findings. Section 6 presents conclusions and outlines future work.

## 2. Background

The growing demand for affordable portable devices has driven the development of BCI systems for accurate neurophysiological data acquisition. Studies emphasize their role in health, neuromotor rehabilitation, cognitive neuroscience, and movement analysis [2,33,34]. Building on this foundation, this section reviews prior work that consolidated BCIs, highlighting their impact on medical research, rehabilitation, and emerging trends in brain activity applications [1,13].

### 2.1. Healthcare and Rehabilitation

Within healthcare and rehabilitation, one of the primary challenges is the high cost and operational complexity of clinical multichannel EEG systems, which significantly restrict their accessibility beyond specialized environments [15]. To overcome this limitation, recent research has focused on the development of portable, wireless, and low-cost solutions capable of detecting both motor imagery states and actual movements [29,35]. Such advancements not only facilitate continuous monitoring of brain activity but also contribute to the design of personalized neuromotor rehabilitation programs.

Previous studies have further shown that the recognition of EEG patterns related to lower-limb motor intention can be translated into control signals within BCI frameworks [7,36]. These signals serve to operate prostheses, exoskeletons, and other assistive technologies, thereby promoting functional recovery and enhancing patient autonomy.

### 2.2. Signal Processing Algorithms

Among the most significant advances in EEG research, several studies emphasize that combining portable hardware with advanced signal-processing algorithms is essential to achieve accurate classification of mental states [29,32,36]. In particular, ref. [29] reports that signal preprocessing paired with machine-learning and deep-learning models such as Support Vector Machines (SVM), Convolutional Neural Networks (CNN), Long Short-Term Memory (LSTM) networks, multimodal fusion strategies and decomposition algorithms, improve the signal-to-noise ratio while preserving the relevant characteristics of EEG signals. Regarding classification, machine-learning approaches show that algorithms such as SVM, Random Forest (RF), and K-Nearest Neighbors (KNN) achieve strong performance in discriminating between cognitive and motor tasks [2,4,29].

Other studies have further validated these methods in practical BCI applications, showing that their ability to generalize across sessions and subjects contributes to more reliable recognition of motor imagery states. These advances highlight the central role of machine-learning strategies in translating EEG patterns into actionable information, thereby reinforcing their importance in the design of portable and low-cost BCI systems.

### 2.3. Trends in BCI and EEG Research

BCI systems have advanced significantly in recent years, particularly in applications involving motor imagery of the lower limbs. The most notable trends include miniaturization and portability. Several studies emphasize that low-cost EEG devices facilitate use in home and community settings, expanding access beyond specialized clinical environments [2,22]. At the same time, other research highlights the benefits of multimodal integration, in which EEG is combined with electromyography (EMG) or inertial measurement units (IMUs) to improve the robustness of motor-intention detection [35,37].

In parallel, another set of studies demonstrates that neural networks and deep-learning techniques for automatic EEG pattern classification provide substantial gains in accuracy and adaptability [29,36,38,39]. These methods are increasingly applied in the development of BCIs for post-stroke rehabilitation, exoskeleton control, and gait monitoring in patients with reduced mobility [1,40]. Overall, these advances underscore the importance of optimization algorithms, robust preprocessing strategies, and technological accessibility in refining BCI systems, thereby accelerating their translation into real clinical applications.

## 3. Materials and Methods

This section presents the methodological approach adopted for the design and validation of the proposed system. The first subsection describes the methods and stages involved in building the motor imagery recognition system, including EEG signal acquisition, processing, filtering, and pattern detection using AI algorithms. The second subsection details the materials, devices, and tools employed in the prototype development, covering both hardware and software components.

Finally, the third subsection explains the experimental procedures carried out in a controlled laboratory environment for the preliminary validation of the system. Together, these materials and methods form the foundation for the empirical analyses presented in Section 4, where the acquisition of EEG data, the construction of the dataset, and the evaluation of system performance are reported in detail.

### 3.1. Methods

The methodological design of this research combined theoretical formulation with practical implementation in a structured sequence. The process began with the identification of the problem and a comprehensive literature review, continued with the conceptual and technical design of the prototype, and advanced through the definition of the experimental protocol and implementation of the system. Data collection was then carried out under controlled conditions, followed by empirical validation and analysis of the results.

Table 1 summarizes the methodological specification of the study, providing a structured view of how the research gap was addressed, how data acquisition and processing were organized, and how these steps connect to the final validation of the prototype. This overview enhances clarity and facilitates reproducibility, serving as a roadmap of the project’s implementation.

The approach described in Table 1 guided the development of the study, ensuring consistency between the conceptual review, experimental design, and empirical validation. By explicitly linking the stages with concrete methodological actions, the table clarifies the rationale of the project and highlights how methodological rigor was maintained across all phases. After defining this framework, the materials required for the system implementation were selected and configured, as detailed in the following section.

### 3.2. Materials

The implementation of the system required the integration of hardware and software components to enable the acquisition, processing, and analysis of EEG signals. Table 2 lists the materials employed, distinguishing between the physical devices used for data capture and management and the software tools that supported development, processing, and visualization.

The system was built around the NeuroSky MindWave Mobile 2, a device recognized for its portability, low cost, and ease of use in non-invasive EEG acquisition applications [41]. It records brain electrical activity through a single frontal channel (FP1) and transmits the data wirelessly via Bluetooth. The selection of FP1 was not discretionary but determined by the design of the NeuroSky Mindwave device, which restricts acquisition to a frontal pole electrode.

Although this site is not physiologically optimal for lower-limb motor imagery, its use reflects the device’s orientation toward low-cost, portable applications with simplified preparation. Within these hardware constraints, the study aimed to evaluate the feasibility of extracting discriminative information for motor imagery classification, thereby assessing the potential of minimal EEG configurations in real-world portable BCI scenarios.

While multi-channel EEG systems provide richer cortical information and greater spatial resolution, their higher costs, longer setup times, and increased preparation complexity limit portability and practical deployment outside controlled laboratory settings. In contrast, a single-channel configuration reduces hardware barriers, enabling rapid use in environments where accessibility and ease of operation are critical. This trade-off motivated the exploration of whether sufficient signal quality could be achieved under simplified conditions.

The processing unit was implemented on an embedded system based on a single-board computer (SBC), specifically the Orange Pi 3B. This compact, low-power microcomputer handled data reception, storage, and processing, while supporting advanced numerical analysis and machine-learning libraries, providing an efficient and robust environment for system development.

The software architecture, implemented in Python 3.10, ensured seamless communication with the EEG device through the MindWave Mobile SDK and supported advanced processing with NumPy, Pandas, and SciPy. These libraries facilitated data structuring and preprocessing, while Scikit-learn enabled the training and validation of classifiers such as SVM, Random Forest, and KNN. Matplotlib was employed for signal visualization and representation of experimental results, complementing the hardware architecture and establishing a robust, flexible, and scalable environment for EEG analysis.

### 3.3. Design and Development of the Prototype

Based on the selected materials, the prototype was designed as a compact system that integrates EEG acquisition and embedded processing. Its architecture was structured on two levels: a physical layer that includes the EEG device and the SBC, and an algorithmic layer that defines the stages of signal analysis.

**Figure 1:**
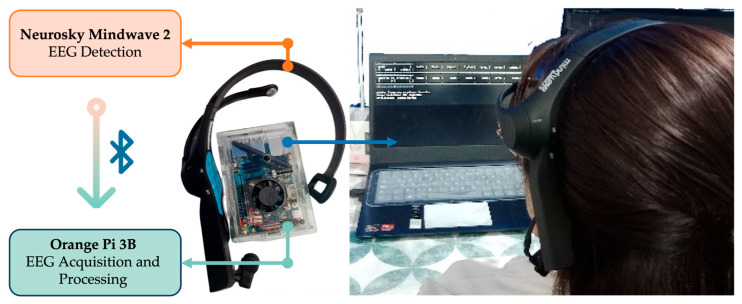
General representation of the hardware layer, integrating the EEG acquisition and embedded processing modules.

The physical layer includes the modules responsible for EEG acquisition and embedded processing. Figure 1 shows the structural diagram of the proposed system, consisting of a single-channel EEG device for recording brain activity and an SBC-based processing unit for storage management and initial processing.

**Figure 2:**
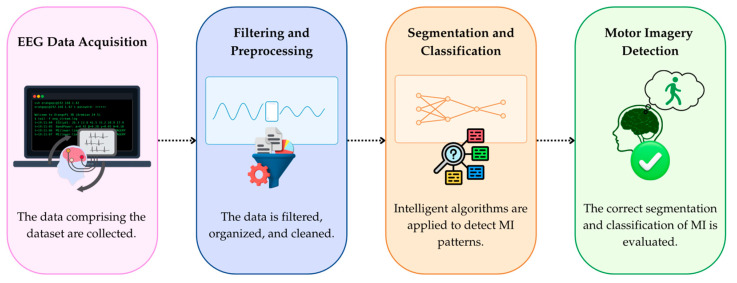
Algorithmic flow of acquisition, filtering, feature extraction, and supervised classification.

The algorithmic layer defines the stages of EEG processing that transform raw data into information suitable for classification. Figure 2 illustrates this workflow, which comprises acquisition, segmentation, filtering, feature extraction, supervised classification, and the generation of final outputs. Data are transmitted and stored in CSV format, serving as the basis for filtering, normalization, and feature extraction. The aim is to identify patterns associated with motor imagery and accurately discriminate between rest, motor imagery, and actual movement in the lower limbs.

**Figure 3:**
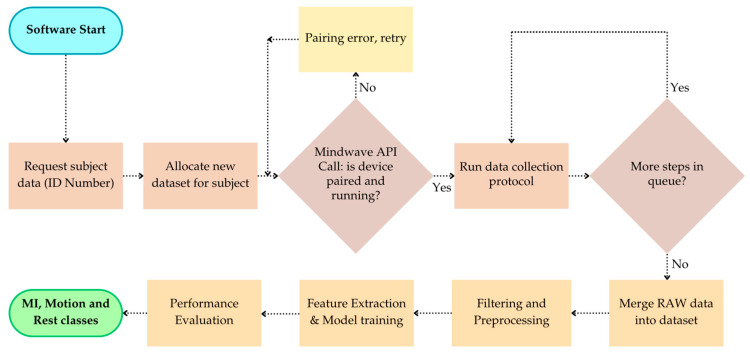
Workflow of the proposed BCI software: subject data registration, device pairing, data acquisition, preprocessing, feature extraction, and supervised classification of MI, motion, and rest states.

The detailed algorithm implementation, illustrated in Figure 3, begins with software initialization, followed by subject data entry (ID number) and dataset allocation. The system then performs a MindWave API call to verify device pairing and operation. In case of error, the process retries.

Once connected, the software executes the data collection protocol, which requires the subject to perform a predefined sequence of motor tasks and motor imagery actions. The system checks whether additional steps remain in the queue. If so, the protocol continues. Otherwise, the session is completed, and the raw data are merged into the dataset.

Subsequent stages include filtering and preprocessing, feature extraction, model training, and the classification of rest, motor imagery (MI), and motion states. This workflow ensures consistent acquisition, preparation, and classification of EEG signals for lower-limb motor imagery recognition. The specific tasks included in the data collection protocol are described in Section 3.4.

Building on the processing flow illustrated in Figure 3, the methodological stages can be organized into a structured sequence that consolidates the main steps of EEG data analysis. These stages encompass the transition from raw signal acquisition to filtering, feature extraction, and supervised classification, culminating in performance evaluation. This sequence, summarized in Table 3, ensures consistency and reproducibility in the detection of neural patterns associated with motor imagery of the lower limbs.

The methodology integrates the hardware, software, and experimental procedures required for the implementation of the single-channel BCI system. By combining the EEG acquisition device, embedded processing unit, preprocessing techniques, and supervised classification algorithms, a coherent and reproducible workflow was established for detecting neural patterns associated with lower-limb motor imagery.

### 3.4. Electrode Placement and Characteristics

**Figure 4:**
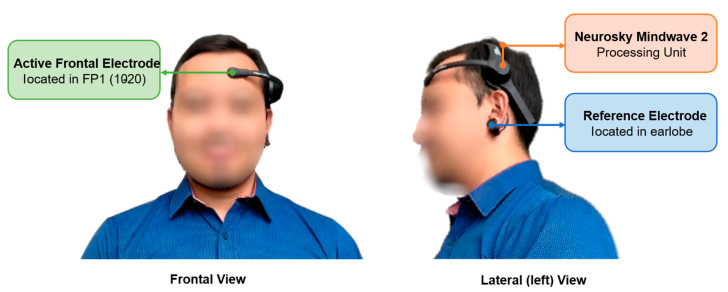
Sensor placement of the NeuroSky MindWave 2 headset: A frontal view showing the Fp1 position, and a lateral view (left) showing the reference ear-clip electrode.

The NeuroSky MindWave Mobile 2 headset is a single-channel (monopolar) EEG device that integrates one active frontal electrode and one reference electrode located on the ear clip. The active electrode is positioned at the Fp1 site, corresponding to the left frontal lobe above the eye, following the international 10–20 electrode placement system. This frontal sensor is mounted on an articulated arm attached to the headband, which applies gentle pressure against the forehead to ensure stable contact. The electrode is a dry stainless-steel sensor with an approximate contact area of 12 mm × 16 mm, providing corrosion resistance, durability, and stable conductivity throughout signal acquisition. The overall configuration and position of both electrodes are illustrated in Figure 4.

The reference electrode (which also acts as ground) is attached to a clip placed on the earlobe. Typically, the left earlobe (A1 site) is used since it offers a neutral, low-noise reference point and comfort for the participant. The ear clip contains a small metallic contact (approximately 8 mm in diameter) that rests against the skin to maintain a reliable electrical connection. Like the frontal electrode, the ear sensor is dry and constructed from stainless steel, eliminating the need for conductive gels and simplifying preparation.

Since dry electrodes inherently have higher skin–electrode impedance than wet ones, the MindWave Mobile 2 employs the ThinkGear ASIC TGAM1 amplifier module, which provides high input impedance and noise filtering to maintain signal integrity even under dry-contact conditions. The headset’s mechanical design maintains an adequate pressure on the sensor (approximately 5.5 kPa/0.8 PSI) to reduce motion artifacts and lower interface impedance. According to the TGAM1 specifications, the analog front-end applies an amplification factor of approximately 2000, band-limits the signal between 3 Hz and 100 Hz, and includes a 50/60 Hz notch filter to suppress power-line interference. The EEG signal is digitized with 12-bit resolution at 512 Hz, providing a measurement range of approximately ±450 µV, which is adequate for scalp-recorded EEG amplitudes in the low-microvolt domain.

As a dry-electrode system, the expected skin–electrode impedance typically ranges from 100 kΩ to 2 MΩ, depending on skin condition, pressure, and preparation. Prior to placement, the skin areas (forehead and earlobe) are cleaned with alcohol or a mild abrasive paste to remove oils and dead cells, improving electrical contact and overall signal quality. For extended recordings, the frontal electrode can be additionally secured with medical tape to prevent slippage and maintain stable contact throughout acquisition.

The system continuously monitors signal quality through a built-in “poor signal quality” indicator, which alerts the operator if contact impedance becomes too high or if excessive noise is detected in the EEG signal. This feedback mechanism ensures the reliability of the acquired EEG data by allowing real-time adjustments of the electrode positioning or pressure.

The described electrode configuration provides a stable, low-noise, and reproducible acquisition setup optimized for frontal EEG recording in portable and low-cost environments. The fixed Fp1–A1 montage, combined with the defined electrical characteristics of the TGAM1 front-end, enables consistent cortical signal capture across participants and sessions. This configuration establishes the hardware foundation for the subsequent data collection protocol detailed in Section 3.5, which specifies the participant preparation, environmental conditions, and acquisition parameters used throughout the experiments.

### 3.5. Data Collection Protocol

Data collection was conducted in a controlled laboratory environment to ensure consistent conditions and minimize the influence of external variables on the recorded electroencephalographic signals.

Ten volunteer subjects participated in experimental sessions that followed a standardized protocol. Each participant received prior instruction on the motor imagery and actual movement tasks to be performed, ensuring correct execution of the procedure. During acquisition, factors such as lighting, noise level, and posture were maintained constant so that the recorded signals would reflect brain activity associated as closely as possible with the proposed tasks.

The overall protocol structure is summarized in Table 4, which describes the main aspects of data collection, including hardware configuration, subject preparation, execution of movement activities, motor imagery tasks, and rest periods between instructions. This organization balanced cognitive load, ensured adequate recovery intervals, and produced a representative dataset for subsequent analysis.

This protocol ensured standardized conditions for all participants, providing a reliable and well-annotated dataset that reflects the neural dynamics of rest, motor imagery, and motion. The resulting data served as the foundation for subsequent processing and classification stages of the proposed system.

### 3.6. Significance and Implications of the Proposed Methodology

The scientific significance of the proposed methodology lies in articulating and implementing a precise applied research question that remains understudied in brain–computer interface research. Specifically, the work examines the extent to which lower limb motor imagery can be decoded when acquisition and processing are constrained by cost, by a single-channel EEG device, and by the need for portability. The contribution is not pursued through a bespoke device or an unprecedented learning algorithm, but through a methodological and procedural framework that can be reproduced and extended. The architecture integrates acquisition, embedded processing, and supervised learning within a coherent pipeline that follows the constraints, materials, and workflow established in the preceding subsections, thereby linking design choices to the practical conditions under which the system is intended to operate.

Within this framework, the decision to work with a frontal single-channel device and an embedded single-board computer is not presented as an optimal neurophysiological configuration but as a realistic operational boundary that many laboratories, clinics, and educational environments face when aiming for rapid deployment, minimal setup, and low barriers to participation. This perspective is especially pertinent for lower limb paradigms, which typically exhibit lower amplitude and discriminability than upper limb activity and therefore require careful attention to data handling even before advanced modeling is considered.

The design gains relevance by linking practical choices with a reproducible methodological strategy. The workflow described earlier specifies acquisition, segmentation, storage in well-defined formats, and construction of a labeled dataset that preserves traceability between experimental conditions and recorded activity. The protocol standardizes preparatory steps, stimulus delivery, and annotations that are needed to later assess the discriminability of resting, imagery, and motion conditions.

In parallel, the methodological plan includes the computation of performance indicators under holdout and cross-validation schemes, along with complementary analyses such as receiver operating characteristics, as outlined in the research stages. This explicit separation between data generation and later analytical choices is central for reproducibility, since it allows independent groups to replicate the pipeline, substitute components, and attribute performance differences to identifiable stages rather than to undocumented implementation details.

The implications of this approach extend to translation beyond controlled laboratory settings. The emphasis on short preparation times, straightforward device operation, and local processing on an embedded platform addresses common constraints in rehabilitation and assistive control contexts where infrastructure and staffing are limited. The protocol favors procedures that can be executed in non-specialized spaces while maintaining consistent quality control over acquisition and annotation.

The result is a prototype that prioritizes deployability and methodological clarity together with accuracy, which is a necessary balance when the target environments demand simplicity and robustness. By documenting the data structures, the labeling conventions, and the evaluation plan, the study provides a practical baseline that can be used to design curricula for training, to support exploratory clinical studies where rapid iteration is required, and to inform early technology adoption in resource-constrained scenarios.

From the perspective of cumulative knowledge, the design helps delineate the trade space among cost, portability, and performance for lower limb motor imagery using minimal acquisition. The work does not challenge the expected advantages of multichannel systems in spatial resolution or robustness to inter-subject variability. Instead, it quantifies and documents how much discriminative capacity can be retained when acquisition is simplified and when processing is executed on accessible hardware.

This information is valuable for designers who must make component choices under budget and usability constraints, for clinicians who need low-burden setups to engage patients quickly, and for educators who require accessible and reliable platforms for teaching and preliminary experimentation.

By making explicit the protocol decisions, the data handling rules, and the evaluation criteria available at this stage of the project, the study contributes validated and transferable methodological guidance that can be extended toward alternative electrode placements, multichannel expansions, or multimodal configurations while preserving transparency and comparability across studies.

## 4. Results

This section reports the performance of the proposed system based on the experimental protocol and predefined evaluation metrics. The analysis focuses on accuracy, reliability, and robustness, highlighting both strengths and limitations observed during validation. In addition, the results are contrasted across different preprocessing strategies and classification algorithms, providing a comparative perspective that situates the system’s capabilities within the broader context of EEG-based motor imagery recognition.

### 4.1. Data Acquisition and Dataset Creation

Recordings were conducted in a quiet, controlled laboratory environment with stable conditions and minimal distractions. Ten healthy adult volunteers (no reported neurological disorders, normal or corrected-to-normal vision, right-foot dominant) participated in the study, each completing five independent sessions according to the protocol described in Section 3.5.

**Figure 5:**
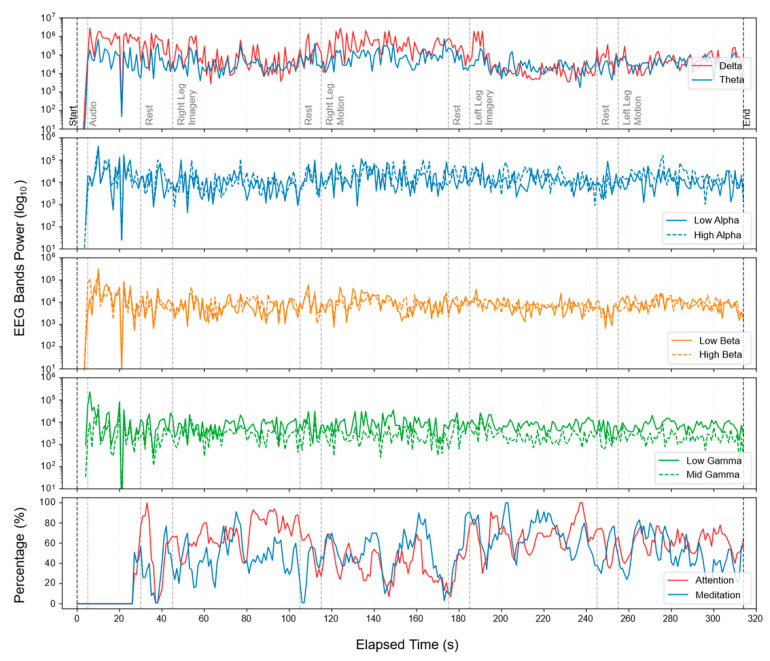
Spectrogram of the raw data recorded during protocol tasks, one subject.

Raw signals were acquired from the frontal FP1 electrode using the NeuroSky Mindwave device and stored in CSV format. Each file contained continuous recordings of brain electrical activity during standardized tasks, together with device-provided cognitive state indicators (attention and meditation). Figure 5 illustrates a representative spectrogram of the raw signal recorded for a single participant during one session.

**Figure 6:**
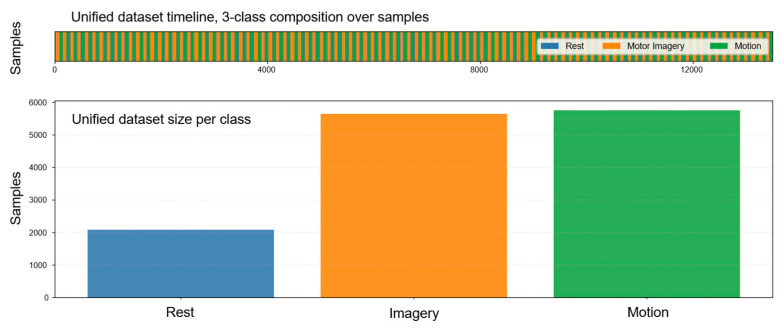
Consolidated dataset overview after merging 50 CSV files (10 test subjects, 5 repetition each one) with task labels and feature annotations.

In total, 50 CSV files were generated (5 repetitions per subject × 10 participants). These files were subsequently merged into a consolidated dataset, as shown in Figure 6, structured into labeled segments corresponding to three task categories: rest, motor imagery, and motion. The target variable was coded in a supervised manner as Target ∈ {Rest = 0, Imagery = 1, Motion = 2}, ensuring reproducibility and traceability between experimental conditions and the corresponding EEG activity.

The dataset preserved the raw time-series signals alongside extracted features, including device-level cognitive indicators (attention and meditation) and relative spectral power across δ, θ, α, β, and γ frequency bands, consistent with the exploratory spectral analysis. This organization provided a balanced and labeled dataset suitable for supervised machine learning, while maintaining the necessary metadata to reproduce experimental conditions.

The consolidated dataset ultimately comprised 14,900 labeled samples. Each record was structured into 12 columns: 10 continuous features (δ, θ, low_α, high_α, low_β, high_β, low_γ, mid_γ, attention, and meditation), one target variable with three categories (Rest, Imagery, and Motion), and one block information column to group contiguous protocol segments. This organization ensured both traceability and consistency between experimental conditions and recorded brain activity.

### 4.2. Preliminary Filter Testing and Selection

To clean EEG signals within the α–β band, zero-phase filtering and smoothing methods were applied. A fourth-order Butterworth bandpass filter (8–30 Hz) with filtfilt was selected for its smooth passband response and steep attenuation, balancing sharp transitions with computational efficiency. This range covers the α (8–12 Hz) and β (12–30 Hz) bands, which are central to motor imagery, and results confirmed high selectivity with strong in-band power and good correlation with reference signals.

The second approach tested was the Savitzky–Golay (SG) filter, designed for temporal smoothing while preserving waveform morphology. An initial configuration with a window of 50 samples and a third-order polynomial produced unstable outputs in short signal segments. To overcome this, a slightly longer window of 75 samples was adopted with the same polynomial order. This configuration minimized error with respect to the reference while preserving oscillatory power and morphology more effectively than long-window SG or simple averaging.

For comparison, two simple smoothing filters were included as baselines. A moving average filter with reflective edge handling was efficient but lacked a defined passband, while a moving median filter reduced impulses and artifacts but flattened the signal when applied as the main stage. A first-order bandpass filter (8–30 Hz) was also used as a performance floor. Quantitative metrics (RMSE, MSE, Bandpower Ratio) are summarized in Table 5.

The first-order bandpass, used as a baseline, obtained a Bandpower Ratio of ≈0.7610 and RMSE ≈ 45,858, confirming its weak out-of-band rejection. The moving average and moving median filters yielded lower RMSE and MSE values, but their Bandpower Ratios were extremely low (<0.05), demonstrating poor preservation of the α–β band. In contrast, the reduced-window Savitzky–Golay filter achieved the lowest RMSE (≈29,255), MSE ≈ 9.554 × 10^8^, and a Ratio of ≈0.6432, highlighting its value as a temporal smoother that minimizes phase distortion while preserving signal morphology. Finally, the fourth-order Butterworth bandpass filter (8–30 Hz) provided the best spectral concentration, with Bandpower Ratio ≈ 0.9988, RMSE ≈ 45,075, and MSE ≈ 2.274 × 10^9^, making it the most effective method for maximizing energy isolation in motor imagery.

Thus, the empirical evidence supports a dual selection: the fourth-order Butterworth bandpass filter as the most effective spectral isolator due to its high energy concentration and low computational cost, and the reduced-window Savitzky–Golay filter as the best temporal fidelity optimizer thanks to its low RMSE and preservation of signal morphology. Both filters were selected to be integrated into the preprocessing pipeline and applied in subsequent experimental stages of the system evaluation, where their spectral and temporal properties also contributed to reducing common artifacts.

As a result, partial attenuation of ocular and muscular artifacts was achieved as an implicit outcome of the preprocessing stage. Low-frequency components typically associated with eye blinks and slow ocular activity (<4 Hz), as well as high-frequency EMG contributions (>40 Hz), were substantially reduced through the combined use of the 8–30 Hz band-pass and the Savitzky–Golay smoothing filter. Although no dedicated EOG reference channel was available, these spectral constraints effectively minimized the most dominant sources of interference in the single-channel configuration, thereby enhancing the reliability of the signals used for subsequent analysis.

Within this framework, the recorded EEG signals, despite being limited to a single channel, provided sufficient discriminative information for machine learning classification once preprocessing and artifact attenuation were applied. Characteristic oscillatory activity within the μ and β bands, consistently linked to motor imagery, could still be captured under this simplified configuration, supporting the feasibility of single-channel input for exploratory BCI applications. Building on this foundation, the next section explores the performance of different supervised classifiers to determine the most suitable model for motor imagery recognition.

### 4.3. Preliminary Classifier Testing and Selection

Five supervised classifiers were trained and compared: Gaussian Naïve Bayes (GNB), K-Nearest Neighbors (KNN), Support Vector Machine (SVM), Convolutional Neural Network (CNN), and Random Forest (RF). These models were selected based on the literature reviewed, as they have consistently shown strong performance in experimental scenarios comparable to this study. The performance of the classifiers, summarized in Table 6, revealed marked differences in their ability to discriminate between the three mental states (Rest, Imagery, and Motion).

The results highlight clear contrasts in classifier performance across the three mental states. Naïve Bayes obtained the weakest outcomes, with overall precision near 43%, confirming its poor suitability for non-Gaussian EEG distributions. KNN performed moderately, yielding F1-scores of 0.55 for Imagery, 0.57 for Motion, and 0.40 for Rest, which indicates more balanced behavior but limited discriminative power. SVM and CNN provided stronger recognition of Motion, both achieving a recall of 0.76, but showed severe limitations in the Rest condition, with recall values of 0.11 and 0.12, and corresponding F1-scores of 0.20 and 0.21. These results suggest that while both models can capture more dynamic patterns, they fail to reliably identify lower-variability states.

Random Forest achieved the most robust and consistent performance. It reached F1-scores of 0.81 for Imagery, 0.81 for Motion, and 0.78 for Rest, outperforming all other models across classes. Unlike CNN and SVM, it maintained high precision and recall simultaneously, showing balanced recognition without major class-specific weaknesses. These findings confirm the suitability of Random Forest for EEG-based motor imagery classification, as it effectively handles nonlinear interactions among features while preserving stable performance across heterogeneous signal conditions.

To contextualize these results, it is important to compare the performance of the proposed system with previous studies. Recent works employing multichannel EEG and advanced classifiers such as SVM, Random Forest, and deep learning architectures have reported accuracies in the range of ≈75–90% for motor imagery recognition tasks [29,36,38,39]. In contrast, simplified or single-channel configurations generally achieve lower performance (≈60–80%) due to reduced spatial resolution [13,22].

Within this landscape, the present system preliminarily achieved an average accuracy superior to 80% with Random Forest, placing it in the competitive range of state-of-the-art methods while relying on minimal hardware resources.

In light of both the internal evaluation and its competitive position relative to state-of-the-art approaches, the Random Forest classifier was selected for implementation in the proposed system, as it consistently achieved the best balance of precision, recall, and F1-score across the three mental states. This model will be integrated into the processing pipeline together with the Butterworth and Savitzky–Golay filters identified in Section 4.2, forming the basis for the subsequent development stages.

### 4.4. Dataset Preprocessing

Once the filtering methods and the best-performing classifier were determined in preliminary testing, the next step was to refine the dataset previously defined in Section 4.1 through a structured preprocessing pipeline. After ingestion from multiple CSV files into pandas dataframes, the integrity of mandatory columns was verified, missing values in EEG signal features were imputed with zeros, and outliers were removed.

The dataframes were then concatenated into a single container (unified_df), preserving float64 types for numerical variables and object type for the block label. This consolidation enabled quality control before modeling while maintaining full traceability of the origin of each sample.

**Figure 7:**
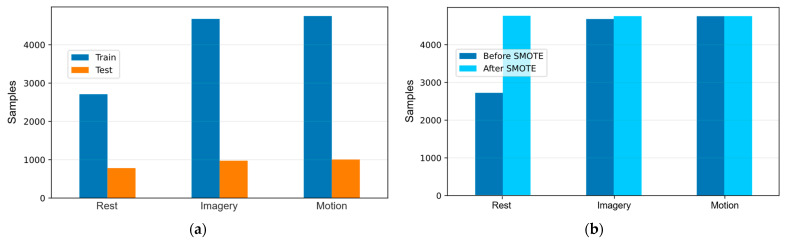
Dataset preprocessing overview. Part (**a**): Holdout split (80% train, 20% test) showing class distributions of Rest, Imagery, and Motion. Stratification preserved proportions, with no rebalancing applied. Part (**b**): Class distributions in the training set before and after Synthetic Minority Over-sampling Technique (SMOTE).

During preprocessing, the dataset was partitioned using a holdout strategy, allocating 80% of the samples for training and 20% for testing. This ensured that performance metrics could be evaluated on data unseen during training, preventing data leakage. In the training split, Imagery and Motion dominated, while Rest remained the minority class. The relative stability between train and test distributions confirmed that stratification worked as intended. As illustrated in Figure 7a, the holdout strategy preserved the original class proportions across both partitions, clearly showing the distribution of Rest, Imagery, and Motion before any balancing procedures were applied.

Class balance was evaluated, and imbalance was corrected only in the training set using the Synthetic Minority Over-sampling Technique (SMOTE) to avoid introducing synthetic instances into external evaluation. Figure 7b presents class frequencies before and after oversampling in the training split. After applying SMOTE, all three classes reached the same sample size, with minimal changes in Imagery and Motion and a substantial increase in Rest.

The procedure corrected the imbalance while preserving the relative structure of the majority classes. Oversampling retains block information and stratification by target, ensuring contiguous protocol segments remain within the same subset and global proportions remain stable. The rebalanced dataset reduced bias toward majority classes and promoted symmetric decision threshold.

The preprocessing pipeline concluded with a balanced training set and an unaltered test set, ensuring fair evaluation conditions. With class distributions corrected through SMOTE and data integrity preserved, the dataset was prepared for supervised model training.

### 4.5. Supervised Model Training

The preprocessed, balanced, and curated dataset was used to feed the processing pipeline developed in previous stages. As part of the preprocessing workflow, three independent versions of the dataset were generated: the unfiltered baseline (normal), a version filtered with the fourth-order Butterworth bandpass (Butterworth), and a version smoothed with the reduced-window Savitzky–Golay filter (Savitzky–Golay). Filtering was applied separately to each dataset block to avoid cross-file contamination and to preserve the integrity of session boundaries.

For each dataset variant, a Random Forest classifier was trained as the final supervised model. The ensemble was configured with 200 decision trees (n_estimators = 200), using log_loss as the splitting criterion to optimize probabilistic predictions. The maximum tree depth was left unrestricted (max_depth = None) to allow the algorithm to adaptively capture complex relationships in the data. A fixed random seed (random_state = 41) ensured reproducibility, while parallel execution across all available processors (n_jobs = −1) accelerated training. This configuration balanced predictive power with computational efficiency and provided a robust framework for EEG-based motor imagery classification.

**Figure 8:**
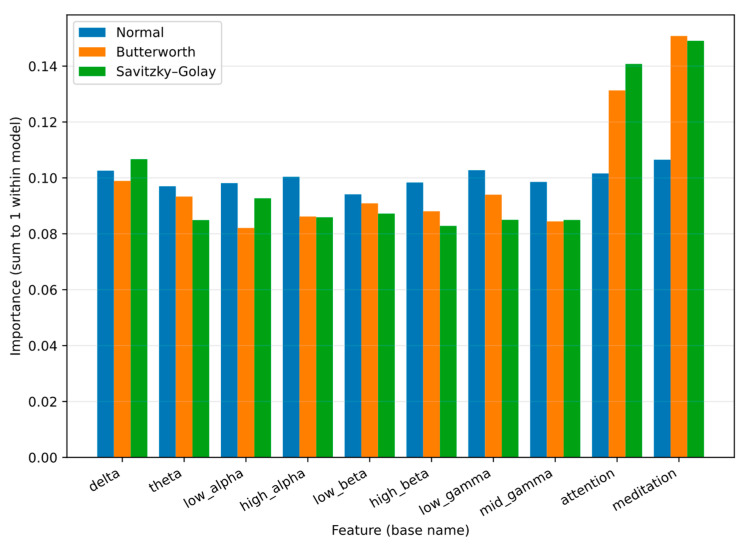
Random Forest feature importance for Original, Butterworth, and Savitzky–Golay datasets, highlighting attention, meditation, and key spectral bands.

Feature importance was computed for each Random Forest model variant to assess the contribution of EEG bands and cognitive metrics to classification. Figure 8 compares the relative relevance of features across three conditions: unfiltered (Original), filtered with Butterworth, and smoothed with Savitzky–Golay.

The results show that attention and meditation consistently dominated as the most discriminative features. Savitzky–Golay filtering further enhanced the weight of meditation, while Butterworth increased the relevance of attention along with low_alpha and theta. Among spectral features, low_gamma and low_alpha emerged as the most informative, with Savitzky–Golay strengthening the contribution of low_gamma and Butterworth slightly improving low_alpha and high_alpha. Theta also gained relevance under Butterworth, whereas delta decreased under Savitzky–Golay. These findings indicate that filtering not only reduces noise but also amplifies specific cognitive and spectral features associated with motor imagery tasks.

### 4.6. Model Performance on the Test Set (Holdout Validation)

The final Random Forest (RF) models, trained with the three dataset variants (Normal, Butterworth, and Savitzky–Golay), were evaluated on the 20% test set reserved during preprocessing and holdout strategy. This assessment provides an unbiased measure of generalization, quantifying how filtering strategies influenced classification accuracy and robustness across unseen data. The performance of the Random Forest models on the holdout set is summarized in Table 7. The metrics reported include overall accuracy, macro-averaged F1-score, and class-specific recall for Rest, Imagery, and Motion across the three preprocessing conditions.

The unfiltered model reached the lowest performance (F1-macro = 0.8025) with weak recall for Rest (0.7187). Applying the Butterworth filter improved the averages (F1-macro = 0.8541) and produced the best recall for Motion (0.8731). In practical terms, these findings demonstrate that preprocessing is a critical stage in BCI systems, as the choice of filter directly affects the reliability of state recognition.

The Savitzky–Golay condition achieved the highest overall results (F1-macro = 0.8671), maximizing recall for Imagery (0.9272) and Rest (0.8708), although Motion recall (0.8067) was lower than Butterworth. These findings indicate that Savitzky–Golay provides the strongest global performance, while Butterworth remains advantageous when the segmentation of the motion class is prioritized.

**Figure 9:**
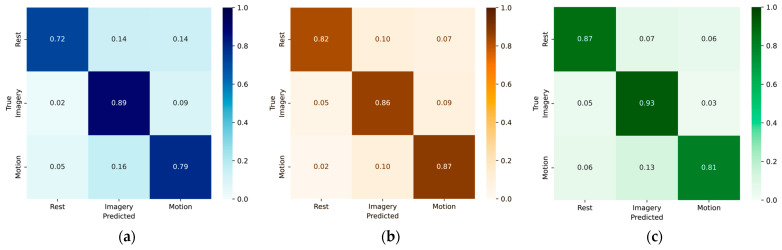
Normalized confusion matrices of the Random Forest classifier on the holdout set: (**a**) unfiltered dataset, (**b**) Butterworth-filtered dataset, and (**c**) Savitzky–Golay–filtered dataset.

In addition to overall accuracy, the system’s discriminative capacity was assessed through confusion matrices. This analysis provides a detailed view of model performance by examining class-specific predictions for Rest, Motor Imagery, and Motion. Figure 9 presents normalized confusion matrices (probabilistic scale) for the three Random Forest variants, enabling a direct comparison of true detections and misclassifications across preprocessing conditions.

Figure 9a shows the baseline confusion matrix for the unfiltered dataset. Rest was recognized with 72% recall, while Imagery and Motion reached 83% and 81%, respectively. Errors were more evenly distributed, with Rest misclassified into both alternative classes and frequent overlaps between Imagery and Motion. With Butterworth filtering (Figure 9b), class-level performance improved. Rest reached 82% recall, Imagery 86%, and Motion 87%. Misclassifications were dominated by Motion–Imagery overlaps (≈10%), while Rest showed more stable recognition with fewer cross-class errors. Savitzky–Golay filtering (Figure 9c) further enhanced Rest and Imagery detection, achieving recalls of 87% and 93%, respectively, but reduced Motion to 81% due to stronger overlap with Imagery (≈13%). Overall, the comparison shows that Savitzky–Golay favors Rest and Imagery separability, while Butterworth achieves a more balanced performance across all classes.

Combining the aggregate metrics from Table 7 with the class-wise recalls in the confusion matrices (Figure 9) provides a consistent picture. The unfiltered model shows the weakest generalization, with broader and more symmetric misclassification patterns. Butterworth improves overall performance but still shows confusions between Motion and Imagery. Savitzky–Golay achieves the best overall balance, enhancing Rest and particularly Imagery separability, although Motion performance decreases. Thus, the matrices corroborate the summary metrics, confirming that Savitzky–Golay provides the strongest support for Imagery segmentation.

**Figure 10:**
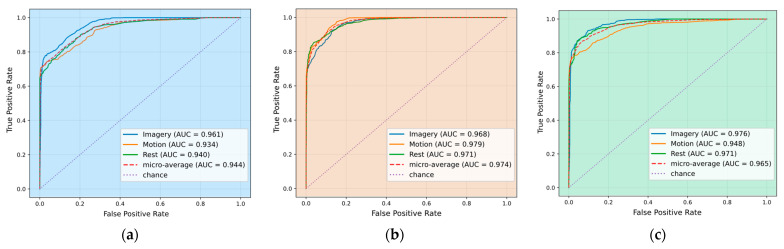
ROC curves of the Random Forest classifier: (**a**) unfiltered, (**b**) Butterworth, (**c**) Savitzky–Golay, showing class-wise separability.

To enhance the class-level analysis provided by the confusion matrices, model performance was further assessed using Receiver Operating Characteristic (ROC) curves and the Area Under the Curve (AUC) metric. This approach captures the trade-off between sensitivity and specificity across decision thresholds, offering a more comprehensive evaluation of discriminative capacity. Figure 10 presents the ROC curves for Rest, Imagery, and Motion under the three preprocessing conditions, enabling a direct comparison of how filtering strategies affect separability in probabilistic terms.

In the unfiltered dataset (Figure 10a), performance is acceptable, but the separation between classes is weaker. Applying the Butterworth filter (Figure 10b) improves overall discrimination, yielding consistently high AUCs across all classes, particularly for Motion, which shows the greatest gain. With Savitzky–Golay filtering (Figure 10c), Rest and Imagery reach their highest AUC values, while Motion remains strong but slightly reduced compared to Butterworth. Overall, these results indicate that filtering improves class separability, with Butterworth favoring Motion recognition and Savitzky–Golay enhancing Rest and Imagery. Both strategies outperform the unfiltered baseline, confirming the impact of preprocessing on classifier discriminative capacity.

### 4.7. Model Performance Under K-Fold Cross-Validation

To complement the holdout evaluation, model robustness was further assessed using k-fold cross-validation. This approach partitions the dataset into multiple folds (k = 5), alternating training and validation subsets to reduce variance in performance estimates and minimize bias associated with a single split. Applying this procedure to the Random Forest models trained on the unfiltered, Butterworth, and Savitzky–Golay datasets provides a more reliable measure of generalization, highlighting the stability of results across different data partitions. The results of this analysis are summarized in Table 8, which reports the average metrics obtained under cross-validation for each preprocessing condition.

The cross-validation results confirm the advantage of preprocessing over the unfiltered baseline. Accuracy improved from 84.50% ± 4.07% (Original) to 87.04% ± 3.71% (Butterworth) and 87.36% ± 4.00% (Savitzky–Golay). A similar trend was observed in macro-F1, rising from 84.15% ± 3.74% to 86.60% ± 3.48% and 87.18% ± 3.81%, respectively. Overall, Savitzky–Golay achieved the highest mean performance, while Butterworth exhibited slightly lower averages but marginally greater stability across folds.

**Figure 11:**
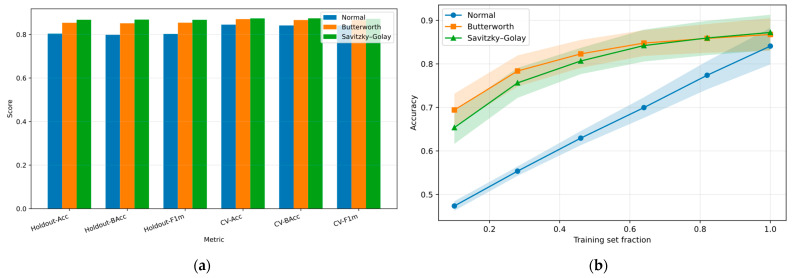
Unified performance analysis of the Random Forest classifier: part (**a**) comparison of holdout and cross-validation (CV) metrics. Part (**b**) unified learning curves under 5-fold cross-validation.

To expand the analysis, Figure 11a compares unified metrics obtained under both holdout and cross-validation schemes, providing a consolidated view of performance across preprocessing strategies. Figure 11b shows the unified learning curves under cross-validation, illustrating how model accuracy evolves with increasing training data and highlighting the stability of the Random Forest classifier across folds.

Figure 11a compares the three preprocessing scenarios (Original, Butterworth, and Savitzky–Golay) across six unified metrics. Accuracy (ACC) refers to the proportion of correctly classified samples, while Balanced Accuracy (BACC) averages recall across classes to compensate for class imbalance.

The holdout metrics correspond to a single train–test split, whereas the cross-validation (CV) metrics represent the same measures averaged over 5 folds. In all cases, Savitzky–Golay achieved the best performance, with Butterworth closely following and the unfiltered baseline consistently lower. The strong agreement between holdout and cross-validation values indicates robust generalization without overfitting, and the narrow gap between ACC and BACC confirms the absence of strong class bias. Overall, preprocessing substantially improved classification, with Savitzky–Golay as the most effective approach and Butterworth as a competitive alternative.

Figure 11b presents the learning curves under cross-validation, showing how validation accuracy evolves as the training fraction increases. The unfiltered condition remains consistently lower, reflecting the impact of noise and limited preprocessing on generalization. In contrast, both Butterworth and Savitzky–Golay enable faster learning and higher accuracy across all sample sizes, with Butterworth slightly ahead in early stages and Savitzky–Golay stabilizing at comparable final values.

These results confirm that filtering not only improves final performance but also accelerates convergence, reducing the amount of data needed to achieve stable accuracy. The overall stability of the curves, together with the progressive gains observed, indicates that the model captures representative neural patterns of Rest, Imagery, and Motion without signs of severe overfitting, while still leaving room for further improvements in generalization for broader applications.

In this context, it is essential to continue refining both signal preprocessing and model configuration. Strategies such as k-fold cross-validation, regularization, and enlarging the dataset could further strengthen system robustness against individual variability and residual noise. The experimental findings therefore validate the technical feasibility of the proposed approach while also pointing to clear opportunities for its evolution into more accurate, adaptive, and clinically applicable BCI systems.

### 4.8. Computational Complexity and Processing Time Analysis

To quantitatively assess the computational cost and runtime behavior of the proposed processing pipeline, a custom-made evaluation framework named “autometrics” was developed in Python. This assert framework integrates multiple open-source analysis libraries to generate a unified report combining static and dynamic metrics. The goal of this framework was to measure code-level complexity, memory efficiency, and runtime distribution across the main stages of signal acquisition, preprocessing, and classification.

The static analysis component relies on the Radon library to compute the Cyclomatic Complexity, Halstead metrics, and Maintainability Index of each module in the implementation. These indicators provide a structural perspective on the algorithmic complexity and readability of the code, highlighting the relative computational weight of each processing stage.

The runtime profiling was performed using cProfile and PyInstrument to record the execution time of each function and visualize the hierarchical call tree of the system. In addition, the Scalene profiler was employed to provide a line-by-line breakdown of CPU and memory utilization, distinguishing between Python-level and native (C-level) execution costs. This multi-layered approach allowed for a detailed understanding of the computational distribution without relying on empirical Big-O approximations, which were not included in this study.

The quantitative results obtained through the developed framework are summarized in Table 9, which consolidates both structural and runtime metrics derived from static and dynamic analyses. This summary provides an integrated view of the algorithmic characteristics, computational load, and system-level behavior of the proposed EEG processing pipeline. The table reports the maintainability and symbolic complexity of the source code (Radon metrics), the cumulative runtime distribution extracted from cProfile, and the fine-grained CPU and memory utilization obtained from Scalene. Together, these indicators allow the identification of dominant computational patterns, bottlenecks related to concurrency or data movement, and the overall efficiency of the implementation in executing preprocessing and classification tasks.

From a structural standpoint, the Radon-based static analysis revealed that the proposed EEG processing pipeline maintains a balanced level of complexity and readability. The Maintainability Index (52.26) situates the implementation within the moderate range, indicating a codebase that is neither trivial nor overly intricate, yet sufficiently organized for iterative refinement and experimental modification. The aggregated Halstead volume (≈1848.96) further reflects a medium symbolic effort, corresponding to a manageable number of operators. The maximum cyclomatic complexity (CC = 7, Rank B) observed in the function apply_filters_to_group corresponds to the expected control branching required by the combined Butterworth and Savitzky–Golay filtering stages. By contrast, auxiliary routines such as cv_by_block, map_target, process_csv, and evaluation functions maintain low complexity scores (CC ≤ 4, Rank A), demonstrating that the computational architecture is dominated by well-encapsulated and linear data-handling components.

The runtime profiling performed with cProfile provided a detailed view of the execution cost of the complete EEG processing pipeline. The total runtime was 336.55 s, distributed across approximately 1.3 million function calls, reflecting the combined activity of data loading, filtering, and model training routines executed under parallel processing. The processing runtime accumulated 314.95 s (93.58%), which corresponds to the main parallel execution block encompassing dataset preparation, filter application, and Random Forest training. The secondary contributor, Sleep time, accounted for 12.85 s (3.82%), representing task scheduling and synchronization within the process pool. All remaining operations, including I/O, feature assembly, and classification calls, consumed only 8.74 s (2.6%) of total runtime. These results indicate that the computational workload is concentrated in the parallelized data-processing stage, while auxiliary tasks introduce negligible overhead.

The line-level profile obtained with Scalene shows bursty CPU use with limited core occupancy. The maximum per-line Python CPU reached ~15.27%, and the maximum per-line core utilization peaked at ~0.64, indicating that compute phases are intermittent and interleaved with coordination/IO rather than sustaining near-core saturation. Memory activity is dominated by NumPy array intermediates: cumulative malloc across instrumented lines is ~542.19 MB, with cumulative growth of ~295.08 MB, and an aggregate copy bandwidth across active lines of ~49.29 MB/s (line-wise aggregates). No GPU activity was detected (CPU-only execution). Taken together, these results confirm that the critical path is the parallel data-processing block, where vectorized filtering produces transient buffers and copies, while overall CPU saturation remains modest due to the cadence of per-file tasks and synchronization intervals.

**Figure 12:**
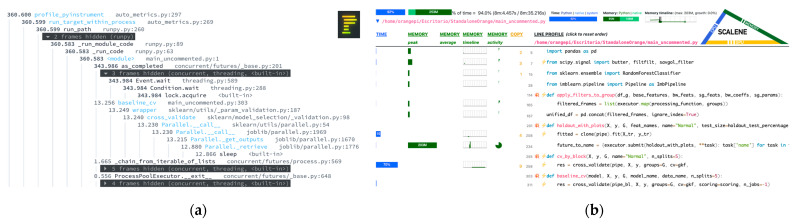
Dynamic profiling of the EEG processing pipeline. (**a**) PyInstrument call-tree report showing execution time distribution across pipeline stages. (**b**) Scalene report illustrating per-line CPU and memory utilization.

Figure 12 illustrates the detailed runtime behavior of the proposed pipeline obtained from dynamic profiling. Part (a) presents the PyInstrument call-tree report, showing the hierarchical distribution of execution time across the main functions of the acquisition, filtering, and classification stages. Part (b) displays the Scalene profile, which decomposes CPU and memory utilization per source line, distinguishing Python-level and native-level execution costs.

Together, these visualizations provide a complementary view of the temporal and computational structure of the pipeline, highlighting where processing time and resource usage are concentrated.

All processing and profiling procedures were executed directly on the embedded platform, ensuring that all reported performance metrics reflect the behavior of the system under realistic operational conditions. The implementation ran entirely on an Orange Pi 3B, a compact single-board computer equipped with a Rockchip RK3566 (Quad-code Cortex-A55) processor (up to 1.8 GHz), an embedded ARM Mali-G52 2EE GPU, 4 GB LPDDR4X RAM, 256 GB eMMC storage, and 32 MB SPI Flash. The device also provides Bluetooth Low Energy (BLE) support, facilitating communication with the Mindwave Mobile 2. These specifications are reported here for reference, as they define the computational environment in which the embedded processing pipeline was executed and can guide future replications or comparative evaluations on similar hardware.

## 5. Discussion and Conclusions

The study of human movement and its associated brain activity has become increasingly relevant in clinical rehabilitation, cognitive neuroscience, assistive robotics, and sports training. Within this context, the present work focused on the design, implementation, and validation of a portable, low-cost BCI system for detecting lower-limb motor imagery.

The prototype was developed using a NeuroSky MindWave Mobile 2 single-channel EEG headset and an Orange Pi 3B embedded unit, with data processing based on classical filtering techniques (Butterworth and Savitzky–Golay) and supervised learning algorithms, primarily Random Forest, to classify Rest, Motor Imagery, and Motion states. The proof-of-concept prototype demonstrated that intelligent algorithms can reliably discriminate neural patterns even from a single frontal EEG channel, in line with recent findings that emphasize the potential of simplified configurations when paired with appropriate preprocessing strategies. Avoiding multichannel headsets reduces experimental complexity and cost, while enhancing portability and usability. These features make the proposed approach practical and accessible for neuromotor assessment in laboratory, educational, and even home-based environments, as supported by current neurotechnology research applied to rehabilitation.

Comparative evaluation under controlled conditions confirmed Random Forest as the most effective classifier, achieving an average accuracy of 87% when combined with Savitzky–Golay (SG) filtering and outperforming alternative models.

This result demonstrates that competitive performance can be achieved using low-cost hardware, supporting the viability of portable BCI systems for future applications in motor rehabilitation and assistive device control. The experimental protocol, which systematically examined EEG acquisition, preprocessing, and classification across three mental states, further highlighted the benefits of SG filtering in improving signal morphology and reducing confusion between Motor Imagery and Motion, a known challenge in EEG-based decoding.

Overall, the findings validate the technical feasibility of the proposed system and establish a solid methodological framework for future developments. Despite the inherent difficulty of distinguishing Motor Imagery from Motion due to overlapping cortical patterns, the system achieved robust discrimination following preprocessing, reinforcing its potential as a real-time neuromotor evaluation tool. Beyond this technical validation, the study also highlights the broader significance of adopting simplified configurations in the context of neural engineering.

The computational cost analysis confirmed that the proposed processing pipeline operates efficiently within the constraints of embedded hardware. The measured execution times, memory usage, and CPU load were all within acceptable limits for real-time or near–real-time EEG processing on low-power platforms. These results demonstrate that the prototype design, both algorithmically and structurally, is well suited for deployment on compact embedded devices such as the Orange Pi 3B, validating its feasibility for portable and cost-effective BCI applications.

In particular, the contribution of this work lies in demonstrating that a single-channel, low-cost EEG device can still achieve acceptable recognition performance for lower-limb motor imagery. This finding underscores the value of simplified approaches in real-world applications, where multichannel systems may be impractical due to cost, setup complexity, or infrastructure demands. Such innovation is important in rehabilitation contexts, where portability and affordability can expand patient access, and for neural engineering research, as it shows that even minimal EEG configurations can provide reliable insights when combined with appropriate processing strategies.

At the same time, certain limitations should be acknowledged. The study was restricted to ten healthy participants, without testing in clinical populations, and relied on the inherent spatial constraints of a single-channel device. Furthermore, the frontal location at FP1 was imposed by the design of the NeuroSky Mindwave hardware rather than by physiological optimization, increasing susceptibility to ocular and muscular artifacts. Although the selected frequency bands and filters contributed to attenuating these sources, the absence of dedicated EOG references or artifact-specific markers remains a limitation.

These considerations emphasize the trade-off between single- and multi-channel systems. While multi-channel configurations enhance spatial resolution and improve classification robustness, particularly for complex or lateralized tasks, they also involve higher equipment costs, longer setup times, and reduced portability. Conversely, the single-channel approach adopted here favors accessibility and ease of use, enabling rapid deployment and potential application in resource-limited environments.

A potential concern is that the 40 Hz binaural tone used as a trial cue could induce entrainment effects within the gamma band. In the present study, this risk was mitigated by excluding the auditory presentation period from all analysis windows. As a result, the features and classification outcomes reported here do not reflect auditory entrainment but instead capture motor imagery and motion-related neural activity. Future studies may further investigate alternative cueing strategies to completely rule out any residual auditory influence.

Looking ahead, addressing these aspects through clinical validation, larger datasets, advanced modeling, and alternative electrode placements such as Cz could further strengthen system robustness and translational potential. In this way, the present work not only highlights a technically feasible solution but also contributes to the growing evidence that accessible and portable BCI solutions can evolve toward more precise, adaptive, and clinically meaningful applications.

## 6. Future Work

Several directions emerge from the present study that could extend the capabilities and applicability of the proposed system. A first line of development involves expanding from the current single-channel configuration to multichannel EEG acquisition. While the present prototype, based on a single frontal electrode (FP1), demonstrated feasibility and competitive performance, the inclusion of additional electrodes would enhance spatial resolution and improve discrimination of cortical activity. Exploring central sites such as Cz, in particular, could provide more direct access to sensorimotor rhythms associated with lower-limb motor imagery.

This extension would be particularly valuable for tasks involving lateralized motor imagery, where richer neurophysiological information could support more precise recognition of movement intention. A multichannel configuration would also increase robustness in clinical and real-world environments, where signal variability and external noise are more challenging.

Another important avenue concerns advanced artifact removal. The filtering strategies implemented here (Butterworth and Savitzky–Golay) effectively improved spectral clarity and provided partial attenuation of low-frequency ocular activity and high-frequency EMG contamination. However, they cannot fully suppress non-neural noise. Techniques such as Independent Component Analysis (ICA) or Ensemble Empirical Mode Decomposition (EEMD) offer greater potential to isolate neural sources and reduce artifacts. Incorporating these methods could yield cleaner datasets, improving both the quality of extracted features and the reliability of classification.

In parallel, the integration of multimodal signals represents a promising path to complement EEG-based detection. Combining electroencephalography with electromyography (EMG) or inertial measurement units (IMU) would provide additional layers of information about motor intention and execution. Such fusion approaches could mitigate the intrinsic limitations of EEG-only systems, enhance robustness against artifacts, and improve classification accuracy. This multimodal framework would also open possibilities for hybrid BCI applications that exploit both central (EEG) and peripheral (EMG, kinematics) indicators of motor activity.

Finally, future work must prioritize validation with clinical populations. The current results, obtained from healthy subjects under controlled conditions, confirm feasibility but cannot be directly extrapolated to patients with neuromotor disorders. Testing with larger and more diverse cohorts, including clinical cases, will be essential to assess translational potential and to adapt the system for real-world neurorehabilitation scenarios. In addition, forthcoming studies will incorporate standardized benchmarking using the MOABB (Mother of All BCI Benchmarks) framework [42], allowing objective comparison with existing motor imagery datasets and pipelines. This will ensure methodological transparency, reproducibility, and positioning of the proposed approach within the broader landscape of BCI research.

Altogether, these lines of research point toward an evolution of the system into a more accurate, adaptive, and clinically meaningful tool. Expanding electrode coverage beyond the FP1 constraint, integrating advanced preprocessing, incorporating multimodal sensing, and conducting clinical validation form a coherent roadmap to strengthen both the scientific contribution and the practical impact of this work.

## Figures and Tables

**Table 1:** Research methodology stages and corresponding activities.

Item	Stage	Description
1	Problem definition and theoretical review	Identification of the research gap in BCI, with emphasis on lower-limb MI and the trade-off between multichannel accuracy and portable feasibility.
2	Conceptual and technical design	Formulation of the hypothesis and design of a portable, low-cost prototype using the NeuroSky Mindwave Mobile 2.
3	Experimental protocol definition	Definition of tasks, lab setup, and recording conditions, including auditory cues and artifact-aware acquisition.
4	Prototype implementation	Assembly and configuration of the device, embedded platform, and software.
5	Data acquisition	Execution of the protocol with healthy participants under controlled conditions.
6	Empirical validation	Application of preprocessing (Butterworth, Savitzky–Golay) and supervised classification models.
7	Analysis and reporting	Evaluation with ROC/AUC and CV metrics, interpretation of results, and documentation of contributions.

**Table 2:** Materials employed for the implementation of the proposed system.

Material	Category	Use
NeuroSky MindWave Mobile 2	Hardware	EEG capture device. Enables wireless acquisition of single-channel EEG signals.
Orange Pi 3B	Hardware	Single-board computer (SBC)-based embedded system. Provides local processing and data storage.
MindWave Mobile SDK library v. 2.0	Software	Enables communication and data collection from the EEG capture device.
Python v. 3.10	Software	The main programming language that enables the development of processing and analysis techniques.
NumPy and Pandas v. 2.3.3	Software	Facilitates numerical processing and structuring of datasets from raw data in CSV format.
SciPy v. 1.16.2 and Scikit-Learn v. 1.7.2	Software	Enables the implementation of the signal preprocessing pipeline and the generation of intelligent classification models.
Matplotlib v. 3.10.7	Software	Enables the visualization of EEG signals and representation of experimental results.

**Table 3:** General stages of the proposed BCI software workflow.

Item	General Description	Algorithm Operation
1	EEG signal acquisition	The acquisition system records the raw signal via a front-end channel before transmitting it wirelessly for local storage and subsequent processing.
2	Filtering and preprocessing	Digital filtering techniques and cleaning procedures are applied to enhance the signal quality and prepare it properly for analysis.
3	Feature extraction	Statistical and frequency-domain variables are extracted to represent the signal in a more discriminative way, thereby improving its clarity and precision.
4	Model training	Machine learning models are trained and validated in order to discriminate between states of rest, motor imagery, and actual motion.
5	Performance evaluation	Performance metrics are calculated to assess the accuracy and robustness of the system in detecting brain activity patterns.

**Table 4:** Experimental protocol applied in ten test subjects, including the phases of motion, motor imagery, and rest periods.

Stage	Technical Objective	Procedure and Key Parameters
Hardware configuration	Establish controlled acquisition conditions	Laboratory setup (EEG device, stimulus display), subject positioning, channel and signal quality verification.
Subject preparation	Standardize initial neurophysiological state	Placement of sensor on FP1 (adjustable band). Pre-task: 40 Hz binaural tones (1 min) *, eyes-open baseline (1 min) for attention calibration.
Experimental paradigm (5 repetitions)	Establish distinguishable EEG conditions (rest, MI, motion, inhibitory control)	Block order: Audio (binaural tones) *, rest, right leg MI, inhibition, right leg motion, inhibition, left leg MI, inhibition, left leg motion.
Data acquisition	Record of EEG target signal	Sampling metrics at 512 Hz: Alpha (8–12 Hz), Beta (13–30 Hz), Gamma (>30 Hz). Sampling metrics at 1 Hz: Attention (0–100); Meditation (0–100) as noise/state control.
Dataset generation	Structure data and annotations for analysis	Series by metric/channel (relative power), condition labels (rest, MI, motion, inhibition), task/event markers, Attention/Meditation, signal quality indicators, and artifacts.

* The 40 Hz binaural tone was employed solely as an auditory cue to signal the beginning of each trial and to standardize participant attention. To avoid potential entrainment effects in the gamma range, the analysis windows were defined to exclude the audio presentation period. Thus, all feature extraction and classification procedures were performed only on EEG segments following the offset of the auditory stimulus.

**Table 5:** Performance of EEG filtering methods in the α –β band (8–30 Hz) evaluated by RMSE, MSE, and Bandpower Ratio, preliminary testing.

Filter	RMSE	MSE	Bandpower RATIO
Butterworth	45,075.34	2.27 × 10^9^	0.9987
Savitzky-Golay	29,255.15	9.55 × 10^8^	0.6431
First-order bandpass	45,858.15	2.37 × 10^9^	0.7610
Moving_median	35,511.05	1.42 × 10^9^	0.0411
Moving_avg	33,261.17	1.24 × 10^9^	0.0341

**Table 6:** Performance of supervised classifiers in discriminating mental states from raw data, preliminary testing.

Classifier	Class	Precision	Recall	F1-Score
Naïve Bayes	All	0.43	0.39	0.41
CNN	Imagery	0.50	0.39	0.44
	Motion	0.44	0.76	0.56
	Rest	0.78	0.12	0.21
SVM	Imagery	0.54	0.47	0.50
	Motion	0.46	0.76	0.57
	Rest	0.91	0.11	0.20
KNN	Imagery	0.51	0.60	0.55
	Motion	0.55	0.58	0.57
	Rest	0.52	0.32	0.40
Random Forest	Imagery	0.78	0.83	0.81
	Motion	0.77	0.84	0.81
	Rest	0.91	0.69	0.78

**Table 7:** Performance of the Random Forest classifier on the holdout set, showing class-specific metrics under normal (unfiltered), Butterworth, and Savitzky–Golay preprocessing conditions.

Model/Dataset	Accuracy Macro	F1-Score Macro	Recall Imagery	Recall Motion	Recall Rest
RF/unfiltered	0.8037	0.8025	0.8872	0.7889	0.7187
RF/Butterworth filter	0.8536	0.8541	0.8585	0.8731	0.8223
RF/Savitzky–Golay filter	0.8673	0.8671	0.9272	0.8067	0.8708

**Table 8:** Performance of the Random Forest classifier under k-fold cross-validation (k = 5), showing class-specific metrics under normal (unfiltered), Butterworth, and Savitzky–Golay preprocessing.

Model/Dataset	Accuracy Macro	F1-Score Macro
RF/unfiltered	0.8450 ± 0.0407	0.8415 ± 0.0374
RF/Butterworth filter	0.8704 ± 0.0371	0.8660 ± 0.0348
RF/Savitzky-Golay filter	0.8736 ± 0.0400	0.8718 ± 0.0381

**Table 9:** Summary of key computational metrics obtained from the developed framework, integrating static code analysis (Radon) and dynamic runtime profiling (cProfile and Scalene).

Aspect	Metric	Value	Interpretation
Static (Radon)	Average Maintainability Index	52.26	Moderate maintainability/complexity.
	Halstead volume	477.65	Medium symbolic/cognitive load.
	Highest cyclomatic complexity blocks (Top 3)	CC = 7 [B] CC = 4 [A] CC = 4 [A]	apply_filters_to_group (Rank B) process_csv (Rank A) cv_by_block (Rank A) Branching concentrated on filtering, processing datasets, and performing cross-validation.
Runtime (cProfile)	Total time	336.554 s	Wall-clock for the full pipeline.
	Function calls	1,298,481 (1,271,753 primitive)	High call volume from pipeline parallel orchestration and library processing.
	Processing time	314.955 s (93.58%)	Total processing time, including dataset loading, filtering, and training of the model, including cross-validation.
	Sleep time	12.855 s (3.82%)	Scheduling latency during task synchronization.
	Other operations	8.744 s (2.60%)	I/O handling, library initialization, and residual computation.
CPU/Memory (Scalene)	Maximum Python CPU per line	≈15.27%	Bursty CPU activity. Workload dominated by concurrent execution phases.
	Maximum Core Utilization per line	≈0.64	CPU cores are used at top performance and 100% capacity under a training random forest model.
	Cumulative Memory Allocations (malloc)	≈542.19 MB	Intermediate NumPy buffers and array copies to RAM.
	Cumulative Memory Growth	≈295.08 MB	Temporary memory expansion during vectorized filtering.
	GPU Utilization	0%	CPU-only processing pipeline.

## Data Availability

The original contributions presented in the study are included in the article; further inquiries can be directed to the corresponding authors.

## Abbreviations

The following abbreviations are used in this manuscript:

ACC	Accuracy
AI	Artificial Intelligence
API	Application Programming Interface
AUC	Area Under the Curve
BACC	Balanced Accuracy
BCI	Brain–Computer Interface
CNN	Convolutional Neural Network
CSV	Comma-Separated Values
CV	Cross-Validation
EEG	Electroencephalography
EEMD	Ensemble Empirical Mode Decomposition
EMG	Electromyography
FP1	Frontal Pole 1 (10–20 EEG electrode position)
GNB	Gaussian Naïve Bayes
ICA	Independent Component Analysis
ID	Identifier
IMU	Inertial Measurement Unit
KNN	K-Nearest Neighbors
LSTM	Long Short-Term Memory
MI	Motor Imagery
MSE	Mean Squared Error
RF	Random Forest
RMSE	Root Mean Squared Error
ROC	Receiver Operating Characteristic
SBC	Single-Board Computer
SDK	Software Development Kit
SG	Savitzky–Golay
SMOTE	Synthetic Minority Over-sampling Technique
SVM	Support Vector Machine
